# The value of arterial spin labelling (ASL) perfusion MRI in the assessment of post-treatment progression in adult glioma: A systematic review and meta-analysis.

**DOI:** 10.1093/noajnl/vdad122

**Published:** 2023-09-30

**Authors:** Tamadur A Alsulami, Harpreet Hyare, David L Thomas, Xavier Golay

**Affiliations:** Department of Brain Repair and Rehabilitation, UCL Queen Square Institute of Neurology, Faculty of Brain Sciences, University College London, London, UK; Department of Diagnostic Radiology, Faculty of Applied Medical Sciences, King Abdulaziz University (KAU), Jeddah, Saudi Arabia; Department of Brain Repair and Rehabilitation, UCL Queen Square Institute of Neurology, Faculty of Brain Sciences, University College London, London, UK; University College London Hospitals NHS Foundation Trust, London, UK; Department of Brain Repair and Rehabilitation, UCL Queen Square Institute of Neurology, Faculty of Brain Sciences, University College London, London, UK; Department of Brain Repair and Rehabilitation, UCL Queen Square Institute of Neurology, Faculty of Brain Sciences, University College London, London, UK; Lysholm Department of Neuroradiology, The National Hospital for Neurology and Neurosurgery, University College Hospitals NHS Trust, London, UK

**Keywords:** ASL, glioma, MR perfusion, pseudo-progression, radiation necrosis

## Abstract

**Background:**

The distinction between viable tumor and therapy-induced changes is crucial for the clinical management of patients with gliomas. This study aims to quantitatively assess the efficacy of arterial spin labeling (ASL) biomarkers, including relative cerebral blood flow (rCBF) and absolute cerebral blood flow (CBF), for the discrimination of progressive disease (PD) and treatment-related effects.

**Methods:**

Eight articles were included in the synthesis after searching the literature systematically. Data have been extracted and a meta-analysis using the random-effect model was subsequently carried out. Diagnostic accuracy assessment was also performed.

**Results:**

This study revealed that there is a significant difference in perfusion measurements between groups with PD and therapy-induced changes. The rCBF yielded a standardized mean difference (SMD) of 1.25 [95% CI 0.75, 1.75] (*p* < .00001). The maximum perfusion indices (rCBF_max_ and CBF_max_) both showed equivalent discriminatory ability, with SMD of 1.35 [95% CI 0.78, 1.91] (*p* < .00001) and 1.56 [95% CI 0.79, 2.33] (*p* < .0001), respectively. Similarly, accuracy estimates were comparable among ASL-derived metrices. Pooled sensitivities [95% CI] were 0.85 [0.67, 0.94], 0.88 [0.71, 0.96], and 0.93 [0.73, 0.98], and pooled specificities [95% CI] were 0.83 [0.71, 0.91], 0.83 [0.67, 0.92], 0.84 [0.67, 0.93], for rCBF, rCBF_max_ and CBF_max_, respectively. Corresponding HSROC area under curve (AUC) [95% CI] were 0.90 [0.87, 0.92], 0.92 [0.89, 0.94], and 0.93 [0.90, 0.95].

**Conclusion:**

These results suggest that ASL quantitative biomarkers, particularly rCBF_max_ and CBF_max_, have the potential to discriminate between glioma progression and therapy-induced changes.

**Key Points**

(1) An appreciable pooled difference was found in blood flow measurements between two groups of true progression and treatment-related effects.
(2) Diagnostic accuracy estimates were relatively high and similar across all ASL-derived quantitative biomarkers.

**Importance of Study**

Distinguishing true progressive disease (PD) from therapy-induced changes has been an extensive research area due to the substantial clinical impact on patient management. While a prompt treatment plan modification is required when glioma progression is confirmed, continuing the standard care regimen will typically accompany a diagnosis of treatment effects. Magnetic resonance imaging (MRI) is the method of choice for glioma post-treatment assessment. However, both PD and treatment-related effects may cause BBB impairment, resulting in similar manifestations on MR imaging, which in turn adds further complexity to treatment response assessment. Therefore, there has been a drive to develop a quantitative monitoring biomarker that is insensitive to BBB disruption and could reliably distinguish PD from therapy-induced changes and, therefore, would have a direct influence on clinical decision-making.

The incidence of brain tumors has increased globally over the past 20 years by more than 40%.^1^ The most prevalent primary intra-axial brain tumor is glioma (>80%), with glioblastoma being the most frequent subgroup (45%). Glioblastomas are associated with limited survival,^2^ despite improvements in treatment plans in recent years.^3^ Presently, maximal debulking surgery, followed by radiotherapy with concomitant temozolomide, and further by adjuvant temozolomide, is the standard care regimen.^4^

Over the last decade, there have been several attempts to distinguish true progressive diseases (PD) from treatment effects because of the substantial potential influence on clinical patient management.^4^ While a prompt modification in treatment strategy with second-line surgery/therapy initiation or ineffective plan termination is required when glioma progression is confirmed,^5^ the standard of care regimen will typically be continued if imaging changes are identified as treatment effects.^4^ For this crucial reason, there has been a drive to develop a quantitative monitoring biomarker that could reliably distinguish PD from therapy-induced changes and, therefore, would impact clinical decisions.

Currently, magnetic resonance imaging (MRI) is the method of choice for glioma post-treatment management, which is largely based on the assessment of signal extent on T2-weighted and FLAIR (fluid-attenuated inversion recovery) images, and the identification of blood–brain barrier (BBB) disruption in the form of contrast enhancement, through the administration of a gadolinium (Gd)-based contrast agent. However, both PD and treatment effects may cause BBB disruption,^6,7^ or changes on T2/FLAIR images, resulting in similar effects on MRI contrast, which in turn adds further complexity to the assessment of treatment response.^8,9^

Arterial spin labelling (ASL) is a perfusion MRI-based technique that uses blood water as an endogenous freely diffusible tracer, unlike other perfusion MRI approaches, such as dynamic susceptibility contrast MRI (DSC-MRI), and dynamic contrast enhanced MRI (DCE-MRI), which require injection of an exogenous contrast agent. ASL was originally proposed and developed in the early 1990s^10^ and has seen a significant increase in interest over the past decade due to concerns with contrast administration, as well as MRI hardware and pulse sequence improvement.^11^ Its non-invasive nature is particularly useful in pediatric populations, patients with impaired renal function and those with difficult intravenous access following chemotherapy. Absolute cerebral blood flow quantification is also feasible with ASL, and as the blood water is a freely diffusible tracer, ASL-derived quantitative biomarkers are less sensitive to BBB disruption than other Gd-based perfusion methods.

Hence, this study aims to systematically review and perform a meta-analysis of ASL-derived biomarker efficacy, including relative cerebral blood flow (rCBF) and absolute cerebral blood flow (CBF), in glioma treatment response assessment. More specifically, discrimination between PD and treatment effects (ie pseudo-progression and/or radiation necrosis) in treated gliomas will be evaluated quantitatively.

## Materials and Methods

### Literature Retrieval

A literature search was performed concerning the role of ASL in post-therapy assessment of gliomas, using sources from Medline, Embase, and Web of Sciences databases, until January 6, 2022. Based on the PICO (Population, Intervention, Comparison, Outcome) approach, the following research question was formulated: “What is the diagnostic value of arterial spin labelling (ASL) in the discrimination of post-treatment progression from treatment induced changes in Adult Glioma Patients?,” and search terms have been identified accordingly: “glioma or glioblastoma or astrocytoma or oligodendroglioma” and “arterial spin or artery spin.” Because studies on the topic are rather limited and to minimize the chances of missing eligible articles, the outcome component terms (ie progression or pseudo-progression or radiation necrosis) were not included in the search. The search performed was restricted on studies published in English Language only.

**Figure 1.**
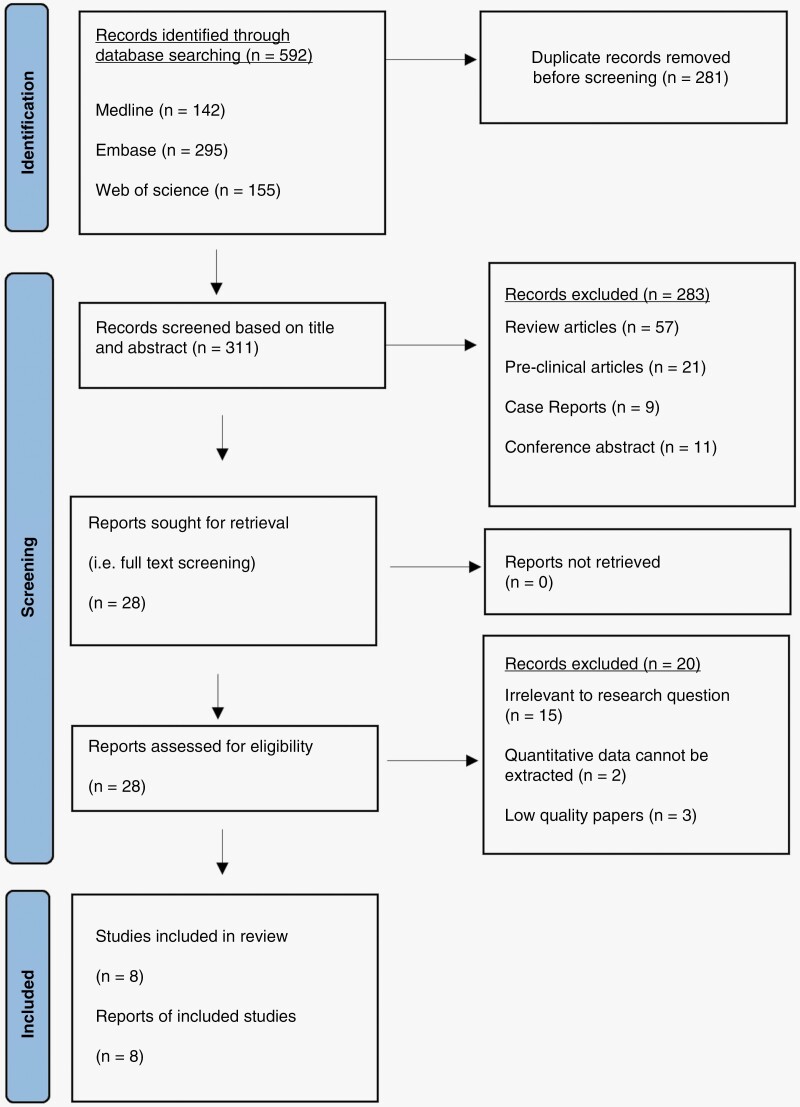
PRISMA flow diagram illustrating identification and selection process.

This systematic review and meta-analysis follow the widely accepted Preferred Reporting Items for Systematic Review and Meta-analysis (*PRISMA*) guidelines.^12^ Initially, a total of 592 records were identified, which were reduced to 311 after duplicate removal. Subsequently, title and abstract screening were performed in order to exclude records that did not match set inclusion criteria, followed by a full text screening of the remaining articles to further exclude irrelevant records. Ultimately, eight eligible studies selected for inclusion were included (Figure 1).

### Literature Inclusion and Exclusion Criteria

Inclusion criteria were: (1) ASL quantitative parameters used to differentiate progression from treatment related changes (ie relative and absolute measures); (2) either pulsed arterial spin labelling (PASL) or pseudo-continuous arterial spin labelling (PCASL) schemes; (3) all subtypes of glioma; (4) studies performed on adult population; (5) consideration of follow-up imaging or histopathology as Reference standard; (6) blood flow measurements are provided on a continuous scale or diagnostic test values can be obtained indirectly or directly in a fourfold table (ie true-positive (TP); false-positive (FP); false-negative (FN); true-negative (TN)).

Exclusion criteria were: (1) unpublished conference abstract and literature duplication; (2) high similarity, such as articles written by the same author and published in the same year; (3) preclinical studies, such as animal model experiments; (4) pediatric studies or those which included metastasis or other primary brain tumors; (5) articles where quality is compromised, with methodological concerns or where identifiable mistakes were present.

### Data Extraction

Basic information was extracted from the literature. This included (but was not limited to) publication year, country, first author’s name, patient’s age, glioma grade, follow-up duration, treatment effect, number of progression and treatment effect cases, scanner field strength, scanner vendor, ASL labelling scheme, and readout sequences. Genetic information (ie O^6^-methylguanine DNA methyltransferase (MGMT) promoter methylation, isocitrate dehydrogenase (IDH) mutation, 1p/19q codeletion) was not provided by the literature. The Web Plot Digitizer was used to extract data illustrated in graphs. When data spread was measured by the interquartile range/range,^13^ the standard deviation (SD) was estimated using method of Wan.^14^

Threshold values were also extracted, with corresponding diagnostic accuracy estimates (ie sensitivity and specificity). RevMan 5.4.1 software (Cochrane, UK) was used to indirectly calculate fourfold table values, using the sensitivity, specificity, and number of PD and treatment-related effect cases provided by the literature.

Two main parameters derived from ASL were of interest in this study: CBF and rCBF. CBF describes perfusion in units of mL/100g/min.^15^ rCBF is calculated as the ratio of the mean blood flow in the lesion region of interest (ROI) to the mean blood flow within a contralateral ROI of normal appearing brain tissue. Due to the inconsistency in ROI definition among records used in this synthesis, renaming of their outputs in this study was crucial. Outlining the tumor entirely on structural MRI scans is thus referred to henceforth as the *mean* absolute or relative blood flow, while a *maximum* measure represents ROI placement over the highest signal on the perfusion map.

### Quality Evaluation

The Quality Assessment of Diagnostic Accuracy Studies (QUADAS-2) tool recommended by Cochrane was adopted as the evaluation basis of both risk of bias and applicability of primary diagnostic accuracy studies.^16^ This involves the assessment of four key domains: (1) patient selection; (2) index test; (3) gold standard; (4) flow and timing. In terms of risk of bias, each of those four domains was evaluated, while assessment of the first three domains was carried out to assess concerns regarding applicability.

### Data Analysis

#### Heterogeneity assessment.

Heterogeneity in this study could arise from variability of several factors among the included studies. These include methodological differences, such as the use of different field strength, labelling scheme, acquisition method, reference area for the normalization, and CBF quantification model, as well as variability in cohort characteristics, such as pathological subtype, follow-up length, age, and gender.

A statistical analysis was performed using RevMan 5.4.1 software. The chi-square test was used to test the hypothesis that all articles measured the same effect, and significance was established as *p* < .05. The percentage of variation in the meta-analysis that can be attributed to heterogeneity was provided by the inconsistency index (*I*^2^). According to Cochrane guidelines, the *I*^2^ statistic can be interpreted approximately as follows:

0% to 40%: insignificant heterogeneity

30% to 60%: moderate heterogeneity

50% to 90%: substantial heterogeneity

75% to 100%: considerable heterogeneity

#### Quantitative synthesis.

Effect size estimation with 95% confidence interval was carried out using RevMan 5.4.1 software (Cochrane, UK). Although the continuous variable outcomes (ie flow measurements) were uniform, they have been measured differently in the eight studies, which in turn could affect the accuracy of the pooled outcome in this meta-analysis. In such circumstances, standardizing the results of each study to a uniform scale before they are merged is necessary. Therefore, to pool an effect estimate across studies, the standardized mean difference (SMD) of blood flow measurements was used, rather than the mean difference (MD). A random-effect model was applied to merge statistics due to observed heterogeneity.

Diagnostic accuracy values (sensitivity and specificity) were modeled jointly using a bivariate model in order to estimate the pooled outcomes with their 95% confidence interval. This approach preserves the two-dimensional nature of diagnostic accuracy and utilizes a hierarchical structure of data distribution in terms of two levels. Within study variability (ie random sampling error) is accounted for at the first level by assuming a binomial distribution for the sensitivity and 1-specificity of each study, respectively. At the second level, between-study variability (ie heterogeneity) is considered by assuming the logit-transformed sensitivity and specificity to have a bivariate normal distribution between studies. The summary receiver operating characteristics (SROC) curve was constructed with the use of the hierarchical summary receiver operating characteristics (HSROC) model, as described previously,^17^ and the corresponding area under curve (AUC) with 95% confidence interval was estimated. This analysis was performed using STATA 17.1 (StataCrop LLC, College Station, TX, USA).

#### Publication Bias Evaluation.

Publication bias assessment was performed using STATA 17.1 (StataCrop LLC). This involves visual investigation of a funnel plot: a scatter plot of study-specific effect estimates versus precision (ie standard error (SE)). In the absence of publication bias, the points (studies) in the funnel plot are expected to form a symmetric inverted “funnel” shape, while asymmetrical funnel plots could indicate publication bias presence. Statistically, asymmetry was tested using the Egger test to examine the association between effect sizes and their measure of precision (effect-size SE). Significance level was set at *p* < .05.

#### Sensitivity analysis.

To assess the stability of the studies included and the impact of a single record on the overall effect estimates, a sensitivity analysis was performed. This involves an elimination of an individual study and estimation of the remaining records’ pooled effect estimates.

## Results

### Literature Retrieval

Eight records, comprising a total sample size of 267 patients with suspected PD post-therapy, met all inclusion and exclusion criteria and, therefore, were included in this synthesis. The characteristics of the studies included are presented in Table 1. Two studies utilized the PASL labelling scheme, while PCASL was used in the remaining six studies. The sample size in each study was relatively small, with a maximum cohort of 69 cases.^18^ Regarding magnetic field strength, 1.5-T scanners were utilized in three studies, while 3.0-T scanners were used in five studies. Clinical–radiological follow-up was used, as the only reference standard without any histological confirmation in one article^19^; in one other article, pathology was used solely for the same purpose^20^, and both reference standards were used in the remaining six studies. In one of the rCBF studies,^21^ data were presented on a continuous scale and, therefore, was included in effect size estimation and forest plot illustrations. Diagnostic accuracy assessment is based on threshold values along with the corresponding sensitivity and specificity, which were provided in one of the CBF studies.^13^ Both representation forms of data ­(continuous flow measurements and diagnostic accuracy estimates) were provided in (3) CBF^18,20,22^ and (6) rCBF studies,^13,18,19,22–24^ which make them eligible for both analyses.

**Table 1.** Overview of studies characteristics included in the meta-analysis to assess the role of ASL in the evaluation of PD post-therapy.

Author and year	Liu et al. 2020^24^	Manning et al. 2020^13^	Ozsunar et al. 2010^23^	Razek et al. 2018^20^	Seeger et al. 2013^19^	Wang et al. 2018^18^	Xu et al. 2017^22^	Ye et al. 2015^21^
Reference standard	Pathology or radiological follow up	Pathology or CR follow up	Pathology and CR follow up	Pathology	CR follow up	Pathology or CR follow up	Pathology or CR follow up	Pathology or radiological follow up
Specific treatment effect	UR	PsP	RN	RN	SD	RN	UR	RN
Treatment	Surgery + (RT or CCRT)	Surgery + CCRT with TMZ	Surgery + PI & PBT	Surgery + RT	Surgery + CCRT with TMZ	Surgery+ RT	Surgery + CCRT with TMZ	Surgery + (RT or CCRT with TMZ)
Diagnostic parameter	rCBF	CBF, rCBF	rCBF	CBF	rCBF	CBF, rCBF	CBF, rCBF	rCBF
Acquisition method	3D- stack of spirals FSE	3D-stack of spirals FSE	UR	2D- single shot EPI	2D-EPI with crusher gradient	3D-stack of spirals FSE	3D-stack of spirals FSE	3D-stack of spirals FSE
Labeling scheme	PCASL	PCASL	PASL-single slice	PCASL	PASL	PCASL	PCASL	PCASL
Scanner vendor and Field Strength	GE 3T	GE 3T	GE 1.5 T	Philips 1.5 T	Siemens 1.5 T	GE 3T	GE3T	GE 3T
Histology	LGG,HGG	HGG (GBM)	LGG(II), HGG (III&IV)	HGG (III &IV)	HGG (III &IV)	LGG(II), HGG (III &IV)	LGG(II), HGG(III&IV)	UR
*n*	30	32	18	42	26	69	29	21
Mean age and/or (range)	PD group: 47.6 ± 11.4 TE group: 40.5 ± 15.0	56 ± 13	42 ± 11 (20–69)	UR	53.6 ± 13.6	PD group: 40.08 TE: group:46.88	47 ± 11	51.3 (32–63)
Study design	P	R	R	P	R	R	P	UR
Follow-up ­duration	≥6 months	≥6 months	≥12 months	(5-11) months	≥ 6 months median:10 months range:(6–15) m	≥3 months	≥6 months	≥11 months

Abbreviations: CR: clinical-radiological; RN: radiation necrosis; PsP: pseudo-progression; SD: stable disease; TE: treatment effect; PD: progressive disease; UR: unreported; *n*: sample size; P: prospective; R: retrospective; CCRT: concurrent chemoradiotherapy; RT: radiotherapy; PI: photon irradiation; PBT: photon beam therapy; TMZ: temozolomide.

### Quality Evaluation

The outcome of the quality assessment, in terms of both risk of bias and concerns regarding applicability, is summarized in Supplementary Figure S1. As sample selection was randomized in most studies (about 88%), the risk of bias of patient’s selection domain was relatively low, despite one study in which inappropriate patient exclusion was not avoided.^22^ Nonetheless, there was potential introduction of bias in the other three domains; index test, reference standard, and flow and timing. Blindness of the index test (ie ASL) to the reference standard was demonstrated in a quarter of included records,^19,20^ but in one study, the lack of blindness to follow-up MRI was specifically declared.^23^ Declaration of ASL blindness to both reference standards (histology and follow-up imaging) was not found in two studies,^22,24^ and only to follow-up imaging in the three remaining studies,^13,18,21^ representing around 63 % of unclear bias of index test domain, collectively. Apart from Manning and colleagues (2020),^13^ information pertaining to the blindness of the utilized reference standard to the index test was not demonstrated in those articles, resulting in 88% of unclear bias in the domain of reference standard. Varying the standard among the recruited cohort in six articles,^13,18,21–24^ and not including all recruited patients in the analysis in two articles^22,23^ has introduced a high risk of bias in flow and timing domain of 75%. The applied standard reference was applied homogenously among patients in only two studies.^19,20^

Evidence of concerns regarding applicability was not observed in the two domains (patient selection, index test), because only adult glioma patients were included in the present study, and ASL was the test under examination. Because PD could potentially be misclassified with pathological confirmation and/or clinical−radiologic follow-up as reference standards, the introduction of high concerns regarding applicability in the third domain (ie reference standard) was inevitable, however.

### Meta-analysis

#### Relative Cerebral Blood Flow (rCBF).

**Figure 2.**
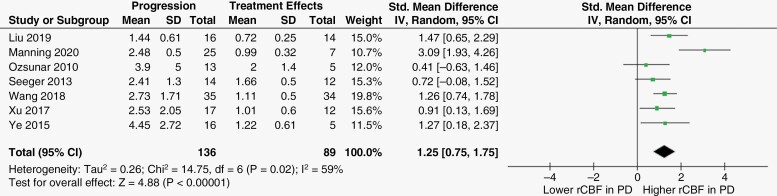
Forest plot graph representing the standardized mean difference in rCBF between progression and treatment effects groups of treated glioma patients. Abbreviations: PD: progressive disease; SD: standard deviation; CI: confidence interval.

Seven studies ^13,18,19,21–24^ evaluated the difference in rCBF measurements between disease progression and a group of therapy-induced changes. The results obtained by the chi-squared test and I^2^ statistic indicated a substantial heterogeneity among records (*p* = 0.02; I^2^ = 59%, respectively), and therefore, a random-effect model was applied to pool an overall effect estimate. A subsequent meta-analysis was performed, which has revealed a pooled effect estimate (ie SMD) for rCBF [95% CI] of 1.25 [0.75, 1.75] (*p* < .00001), showing a statistically significant difference in rCBF measurements, with higher rCBF in the PD group. Figure 2 illustrates the forest plot graph, study-specific effect estimates, and pooled effect estimate of rCBF across the included records.

**Figure 3.**
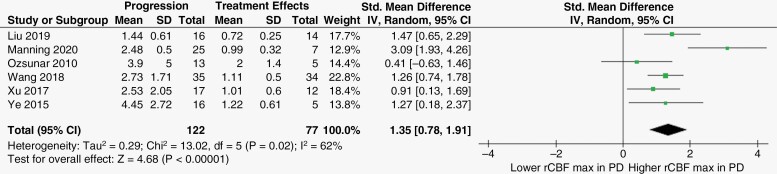
Forest plot graph representing the standardized mean difference in rCBF_max_ between progression and treatment effects groups of treated glioma patients. Abbreviations: PD: progressive disease; SD: standard deviation; CI: confidence interval.

Of the seven articles mentioned above, six^13,18,21–24^ also assessed the discriminatory ability of rCBF_max_. Likewise, the observed heterogeneity was substantial according to chi-squared test (*p* = 0.02) and *I*^2^ index (62%), requiring analysis with a random-effect model. rCBF_max_ was significantly higher in the PD group than in the treatment effects group, with a SMD [95% CI] of 1.35 [0.78, 1.91] (*p* < .00001). Figure 3 represents the forest plot illustration, study-specific effect estimates and pooled effect estimate of rCBF_max_ across the included records.

#### Absolute Cerebral Blood Flow (CBF).

**Figure 4.**
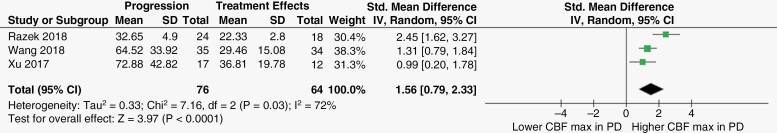
Forest plot graph representing the standardized mean difference in CBF_max_ between progression and treatment effects groups of treated glioma patients. Abbreviations: PD: progressive disease; SD: standard deviation; CI: confidence interval.

Due to the consensus in the current literature in ROI location, which was positioned over the highest signal on perfusion map,^18,20,22^ the difference in CBF_max_ between the targeted groups was the outcome under assessment in the present meta-analysis. In concordance with the rCBF_max_ results presented above, a SMD [95% CI] of 1.56 [0.79, 2.33] (*p* < .00001) was obtained for CBF_max_, indicating a significantly higher blood flow in glioma progression patients than in treatment-related effects cases. This pooled effect estimate was obtained with the use of the random-effect model to merge three articles. Apparently, model selection was attributed to the existence of a substantial heterogeneity among included records, as confirmed by the chi-squared test (*p* = 0.03) and I^2^ index (72%). Figure 4 represents the forest plot, study-specific effect estimates and pooled effect estimate of CBF_max_ across included studies.

### Diagnostic Accuracy

**Figure 5.**
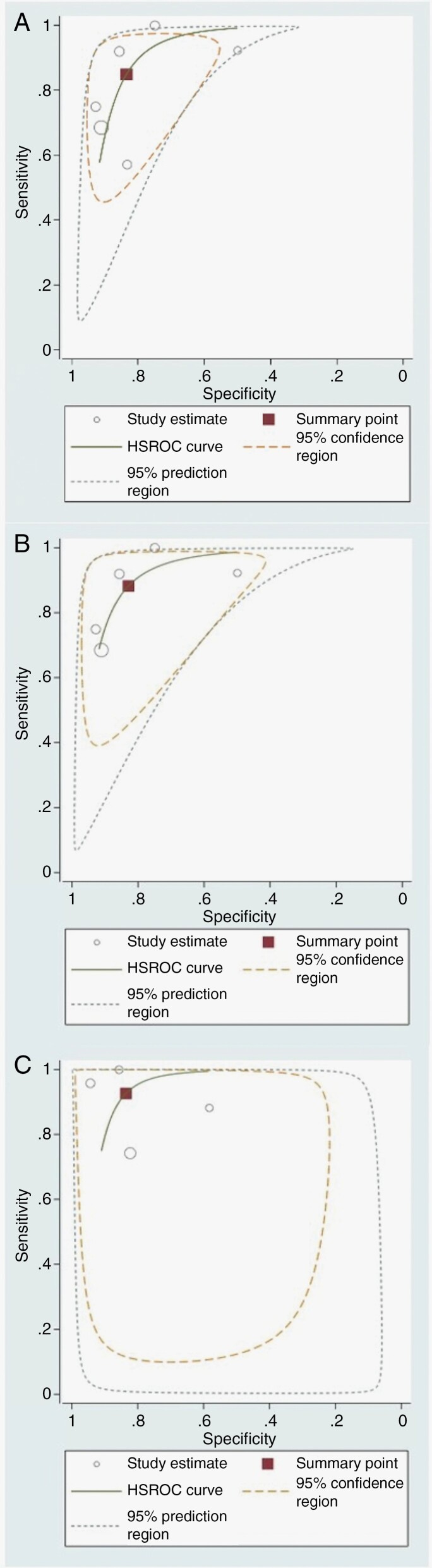
HSROC curve of ASL-derived biomarkers in the assessment of glioma post-treatment progression in adults: (A) rCBF; (B) rCBF_max_; (C) CBF_max_. The squares represent the derived summery points of sensitivity [95% CI] and specificity [95% CI] as follows: (a) 0.88 [0.71, 0.96] and 0.83 [0.71, 0.91]; (b) 0.85 [0.67, 0.94] and 0.83 [0.67, 0.92]; (c) 0.93 [0.73, 0.98] and 0.84 [0.67, 0.93], respectively.

Diagnostic test accuracy evaluation supported the findings of the meta-analysis. The derived summary point of sensitivity and specificity were relatively high for ASL-derived biomarkers in the discrimination between disease progression and treatment-related effects. More specifically, the sensitivity [95% CI] was slightly higher for rCBF_max_ compared to rCBF (0.88 [0.71, 0.96] vs. 0.85 [0.67, 0.94], respectively), but the highest sensitivity was yielded by CBF_max_ (0.93 [0.73, 0.98]), although the improvement in sensitivity was not significant. The specificity [95% CI] was nearly uniform across different ASL metrices, and they were as follows: 0.83 [0.71, 0.91]; 0.83 [0.67, 0.92]; 0.84 [0.67, 0.93], for rCBF, rCBF_max_, and CBF_max_, respectively (Figure 5). Supplementary Table S2 summarizes the diagnostic test accuracy assessment in terms of the estimated points of sensitivity and specificity for different ASL indices.

Nevertheless, a summary line (ie curve) would be more representative for the widely heterogenous data. Rather than a summary point, the trade-off between sensitivity and specificity for different cutoff values reported by different studies can be explained on average. Overall, the area under the HSROC curve was also somewhat high for various ASL-derived biomarkers (ie AUC [95% CI] is 0.90 [0.87, 0.92] for rCBF; 0.92 [0.89, 0.94] for rCBF_max_; and 0.93 [0.90, 0.95] for CBF_max_). These results are illustrated in Figure 5.

Supplementary Table S1 provides a full depiction of the literature proposed cutoff values, four-fold table values and corresponding diagnostic accuracy estimates in distinguishing post-therapy progression and treatment effects of adult glioma.

### Publication Bias

**Figure 6.**
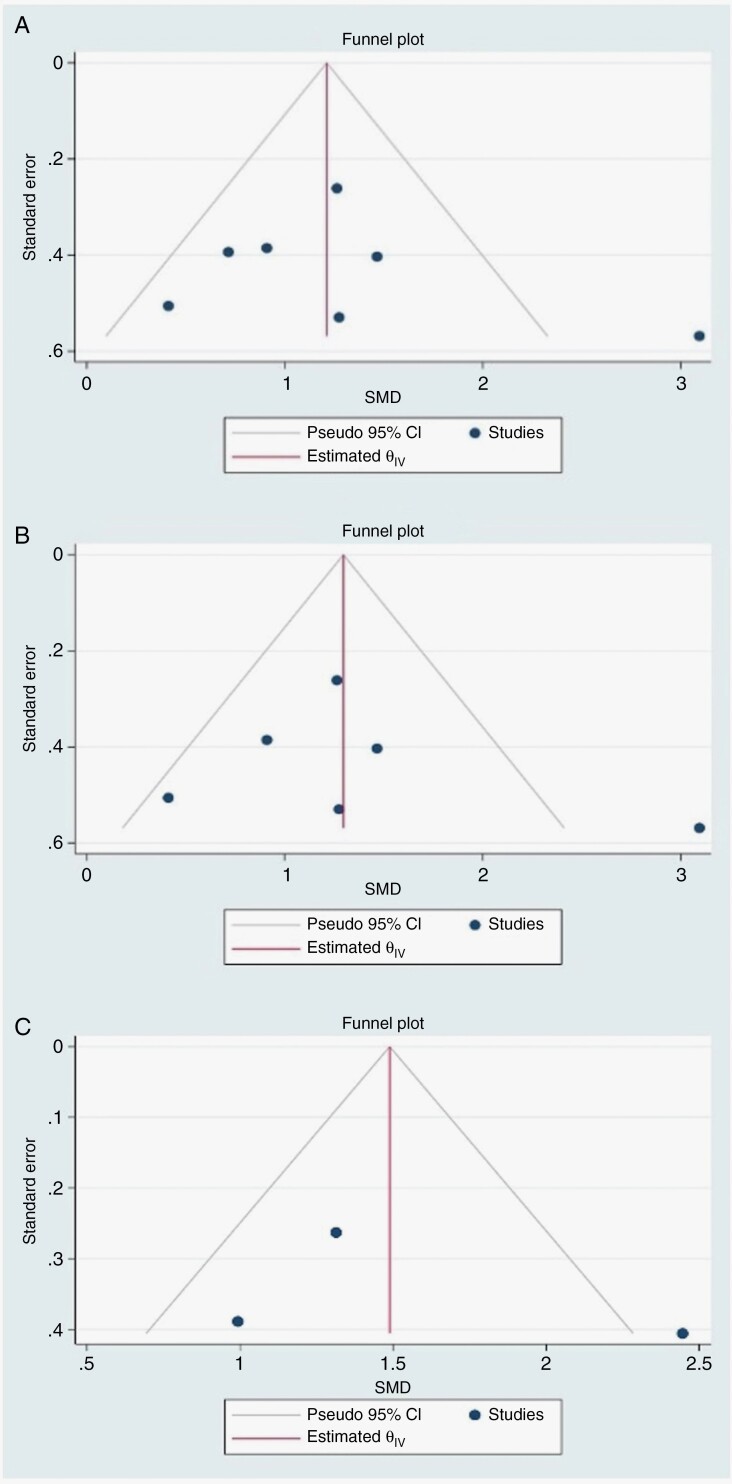
Funnel plot of publication bias in (A) rCBF, (B) rCBF_max_, (C) CBF_max_ studies.

Funnel plots were nearly symmetric about the pooled effect estimate, suggesting no significant publication bias for all ASL biomarkers, whether relative or absolute. This has been confirmed statistically with the use of the Egger test which demonstrated an insignificant association between effect sizes and their SE (ie *p* = .38; *p* = .45; *p* = .65, for rCBF, rCBF_max_, CBF_max_, respectively). Funnel plots for various ASL-derived biomarkers are illustrated in Figure 6.

### Sensitivity Analysis

Effect estimates remained nearly unchanged, with no significant differences among the repeated meta-analyses. This is suggestive of a roughly equivalent influence of the included studies on the estimated overall SMD. More details regarding the sensitivity analysis for the biomarkers obtained by ASL can be found in Supplementary Tables S3–S5.

## Discussion

Distinguishing progressive glioma from therapy-induced changes after treatment has been an extensive research interest area, spanning various modalities of imaging. While previous studies have evaluated CT and MRI for discrimination of radiation necrosis from tumor progression,^25^ or DSC/DCE perfusion measures,^26^ this is the first systematic review to include both early and late treatment-related effects focusing specifically on ASL-based perfusion MRI. This study has demonstrated an appreciable pooled difference in blood flow measurements between two groups of true progression and treatment effects, both with absolute and relative CBF measures. Overall, it was observed that diagnostic accuracy estimates were relatively high and similar across all obtainable quantitative biomarkers by ASL-based perfusion MRI.

In terms of both applications and technical innovation, the field of ASL has been evolving rapidly.^27^ By using blood water as an endogenous diffusible tracer, CBF can be measured non-invasively. However, being an inherently low signal-to-noise ratio (SNR) technique, scan protocol optimization is essential. This is primarily because the inflowing labelled blood constitutes only about 1% of the overall brain tissue signal,^28^ making the ASL signal relatively subtle.

This quantitative synthesis indicates that blood flow measurements were significantly higher in PD patients compared to treatment-related effects group. This could be underpinned by different physiological processes, separating the two responses. While progression is associated with neo-angiogenesis and, therefore, increased hemodynamic activity, reduced perfusion as a result of therapy-induced vascular endothelial damage and coagulative necrosis are associated with therapy-related effects.^29,30^ The effect size of rCBF_max_ was slightly larger than that of rCBF (SMD [95% CI] of 1.35 [0.78, 1.91] compared to 1.25 [0.75, 1.75]). This is anticipated as high-grade glioma tends to be heterogenous, and the most anaplastic part would be more represented by the measure’s maximum. Although not significant, the highest discriminatory ability in the current study was obtained by the absolute flow metric (ie CBF_max_), with a SMD [95% CI] of 1.56 [0.79, 2.33] (*p* < .00001). This has also been emphasized by the diagnostic test accuracy assessment, when the pooled sensitivity, specificity, AUC values were all slightly higher with the absolute measure than with the normalized flow metrices, which is in line with the previously reported findings by Manning and colleagues (2020).^13^

However, given the presence of statistically significant heterogeneity, the quantitative characterization of post-therapy lesions by ASL in the current literature has several limitations in terms of the generalizability and reproducibility. To begin with, glioma grade, applied reference standard, and follow-up duration have not only introduced between study heterogeneity, but within a single experiment, a mixed cohort of high- and low-grade glioma patients were studied,^18,22–24^ and their treatment response was classified based on different standard reference,^13,18,19,22–24^ after variable periods.^13,18–24^ Differences in treatment regimen, the software used for post-processing, scanner manufacturers, and radiofrequency (RF) head coils could also have a considerable impact on the resulting ASL scans. From an image acquisition perspective, important sources of heterogeneity were the applied labeling approaches, readout sequences and the wide range of the post-labelling delay (PLD) times used. Sensitivity and specificity were 0.53^19^ and 0.50,^23^ respectively, at a field strength of 1.5 T, and were higher at 3T^13,22^ due to the higher intrinsic SNR and longer tissue T1 values (blood, healthy brain parenchyma, and tumor).

The unreliability of the histopathological confirmation as a reference standard could be due to two main reasons.^31^ First, because the entire enhancing tissue may represent a mixture of therapy-induced changes and tumor, there is a potential for biopsy sampling bias.^32^ Second, given the background of extensive post-therapy related changes, there is a lack of pathological standardization causing a variety of interobserver diagnostic interpretations.^33^ Nevertheless, it was pragmatically included as an acceptable reference standard in the absence of more accurate available reference standards. Compared to follow-up imaging, it appears to be a more reliable reference standard.^34^

Contrast enhancement on conventional follow-up imaging, on the other hand, appears to be biologically non-specific, which can result in false positive, false negative, and indeterminate outcomes.^34^ False-positive progression and false-negative treatment response could manifest as an increase or a decrease in the enhancing lesion volume, respectively, two distinct scenarios where the lack of specificity of follow-up imaging, and therefore, its limitation as a reference standard, can be demonstrated. In addition, the definition of PD versus treatment effects in the current literature was based on variable frameworks; from the Macdonald criteria^21,35^ to the most recent RANO framework^19,22,24,36^ and later modification (mRANO criteria^13,37^).

The early therapy-induced effect, pseudo-progression, characteristically appears within a period of 6 months after radiotherapy and concomitant temozolomide completion,^4^ and it has been shown previously that 30% of cases appear after the first 3-month period.^38^ However, one study^18^ used a minimum follow-up period of 3 months, which potentially could compromise classification accuracy. Indeed, consideration of appropriate timing of follow-up imaging is necessary in study design.

Absolute blood flow measurements obtained using the same labeling and acquisition approaches in different studies are occasionally conflicting. The cutoff value of 64.2 ml/100g/min reported by Manning et al. (2020)^13^ is significantly higher than that in other studies (32.33 and 36.86 ml/100g/min).^18,22^ Such rather contradictory results are conceivably attributable to various factors. First, a homogeneous cohort of glioblastomas were included, whereas other studies were carried out in a mixed cohorts of both high- and low-grade glioma patients.^18,22^ As has been shown previously, the difference between both grades does not only involve pseudo-progression incidence^39^ and conventional contrast enhanced MR patterns,^40^ but most importantly, the baseline of ASL-derived measurements could be much lower in the case of glioma with lower grade.^41–43^ Secondly, a longer PLD of 2025ms was used in one study,^13^ as opposed to 1525ms used by others.^18,22^ According to consensus recommendations for standardized ASL imaging protocols in clinical trials, a PLD of 2000 ms was recommended for adult clinical patients,^27^ although it should be noted that this recommendation was made in the context of dementia rather than patients with brain tumors.

Similarly, the variability in brain tissue type used for normalization has limited the comparability of relative flow measurements across studies. Seeger et al. (2013)^19^ found that a threshold rCBF value of 2.18 could distinguish between PD and stable disease (SD), but with a non-significant discriminatory ability (*p* = .063). However, the reference region used in this study was the normal appearing white matter (NAWM), and the study was performed at 1.5-T, which makes the inherently low ASL signal more unreliable and problematic to quantify precisely. In brain tumor patients, NAWM could be possibly affected by mild structural axonal fiber loss and demyelination after radiotherapy^44^ and often has a considerably higher water content compared to healthy controls.^45^ Also, CBF of white matter could be underestimated by ASL because of the long transit times, particularly when a short PLD is used.^11,46^ Lower cutoff values have been reported, ranging from 1.11^22^ to 1.57,^13^ using a corresponding PLD of 1525 ms to 2025ms, when the contralateral normal appearing brain tissue was considered as a reference area for normalization, without tissue type specification (ie grey matter (GM) or white matter (WM)). One study^18^ has compared ASL performance to positron emission tomography (PET), and therefore, perfusion analysis used the cerebellum as a reference region for ratio estimation, with a threshold value being specified at 1.86. Consequently, the pooled outcome of this methodological heterogeneity displays a wide spectrum of optimal cutoff values, which in turn has limited the feasibility of finding a clinically meaningful single threshold that could discriminate between progression and treatment-related effects.

Moreover, quantification in the current literature is mainly based on the operator-dependent ROI approach. An alternative approach, known as histogram analysis, could capture tumor heterogeneity and offer a more comprehensive approach with better interobserver agreement, sensitivity, and negative predictive value.^47^ However, regardless of the high user dependency of the ROI method, manual delineation is more feasible in clinical practice than histogram analysis, unless the latter can be seamlessly integrated into a clinical workflow.

Radiation necrosis and pseudo-progression (PsP) have occasionally been considered grouped together and referred to as “treatment effects,”^22,24^ although in fact they can differ in prognosis, histopathology, physiology, and timing.^48^ While the former typically occurs 9 to 12 months or possibly several years after treatment,^49^ the latter could be better defined as new or increased enhanced lesions on structural MRI, typically within 3 to 6 months post-therapy, followed by an improvement or resolution spontaneously.^8,50^ PsP is pathopysiologically distinct from radiation necrosis and most probably caused by endothelial cell injury, which causes tissue inflammation and vascular endothelial growth factor (VEGF) upregulation, leading to edema and increased vessel permeability.^8^ By contrast, more severe injury related to endothelial cell death, autoimmune mechanism, and oligodendrocyte injury could accompany radiation necrosis, leading to a more irreversible fibroid necrosis.^51–53^ A further distinction is that PsP seems to have a more favorable prognosis^8^ and is considerably correlated with MGMT promoter methylation.^4^ Among the studies included in this synthesis, there was no consistency in terms of which treatment effect being evaluated. One of the studies evaluated PsP in glioblastoma patients,^13^ while another assessed radiation necrosis in high-grade gliomas (HGGs)^20^ and two studies combined both treatment effects.^22,24^ In such circumstances, it would not be possible to draw firm conclusions at subgroup level. The difference between these therapy-induced changes in terms of the derived perfusion measurements has rarely been studied; however, the largest anticipated difference between both would be the reduced perfusion in radiation necrosis, as compared to that in PsP. Pseudo-response is a radiological phenomenon whereby treatment with anti-angiogenic agents can produce dramatic and early reduction of tumor enhancement,^54,55^ though the tumor remains stable or even grows over time.^56^ To the best of our knowledge, the clinical value of ASL in the assessment of such response has not been studied to date and, therefore, was beyond the scope of this review.

The usefulness of ASL has been previously studied through several comparisons with other advanced imaging techniques, such as diffusion-weighted imaging (DWI),^20,24^ magnetic resonance spectroscopy (MRS),^19,24^ and more frequently with the well-established contrast-based perfusion methods, DSC and DCE.^3,13,18,19,21–23,57^ This was primarily achieved in terms of the quantitative measurements derived from both techniques which were closely correlatedand the diagnostic accuracy. However, these studies were performed on somewhat small cohorts, and the impact of the utilized software package and postprocessing method were not considered.

The interpretation of the results of this work carries with it various limitations. To begin with, this work was based on a limited number of studies, comprising relatively small cohorts. Large scale prospective studies are, therefore, needed for verification before implementation into clinical practice can be envisaged. However, this study is summarizing the current state of the literature, and the positive results presented here provide good motivation and promising avenues for such studies. A further major drawback is that a wide spectrum of heterogeneity has been observed in the present study, ranging from technical to clinical factors, across a limited number of included studies. This consequently has hindered the feasibility of subgroup analysis and the use of a fixed-effects model. In particular, glioma grades varied not only between studies, but also the majority of these studies were performed in a mixed cohort of high- and low-grade glioma patients. Compared to gliomas of higher grade, derived perfusion measurements from DSC and ASL are much lower in low-grade gliomas (LGGs),^41–43^ where pseudo-progression incidence,^39^ enhancement patterns,^40^ and treatment regimens are also distinct from those of HGGs. This heterogeneity in patient population could limit the generalizability of the obtained results or be a potentially confounding factor. Given the variety of ways in which ASL can be implemented, and the associated effect on the quantitative values generated, the published literature presents a rather heterogenous picture in terms of the threshold values defined. However, the primary aim of this work is not to define a specific threshold value; rather is to collate the currently available evidence regarding the value of using ASL to differentiate tumor progression and treatment effects, to provide an indication of whether is likely to be a fruitful avenue for further investigations. If so, it should encourage further work to explore the optimal ASL sequence and protocol implementation to provide best sensitivity and specificity. Other sources of heterogeneity could remain due to various unidentified factors, including magnetic field inhomogeneity (which can affect ASL labeling efficiency and image quality) and patient movement. Finally, although both histopathology and follow-up imaging were considered as reference standards in this work, they certainly provide inequivalent approaches for reliability.

In this work, the value of ASL-derived biomarkers in the discrimination of PD and therapy-induced changes were evaluated, providing a solid foundation for future investigative studies. This study has demonstrated an appreciable pooled difference in blood flow measurements between two groups of patients with true progression and treatment-related effects, both with the absolute and relative measures. Overall, it was also observed that diagnostic accuracy estimates were relatively high and similar across all obtainable quantitative biomarkers by ASL-based perfusion MRI. It appears, therefore, that ASL-derived biomarkers, particularly CBF_max_ and rCBF_max_, have the potential to discriminate between disease progression and therapy-induced changes in gliomas. Nevertheless, consensus standardization and further investigation are of paramount importance before any widespread quantitative strategy can be implemented.

## Supplementary Material

vdad122_suppl_Supplementary_Figures_1_Tables_1-5
