# Supplementary material for: The value of arterial spin labelling (ASL) perfusion MRI in the assessment of post-treatment progression in adult glioma: A systematic review and meta-analysis
Source: Neurooncol Adv. 2023 Sep 30;5(1):vdad122. doi: 10.1093/noajnl/vdad122 (PMC10576519; doi:10.1093/noajnl/vdad122)
Supplement: vdad122_suppl_Supplementary_Figures_1_Tables_1-5 [file vdad122_suppl_supplementary_figures_1_tables_1-5.docx]

**Supplementary Figure 1. Quality evaluation assessment in terms of risk of bias and concerns regarding applicability.**


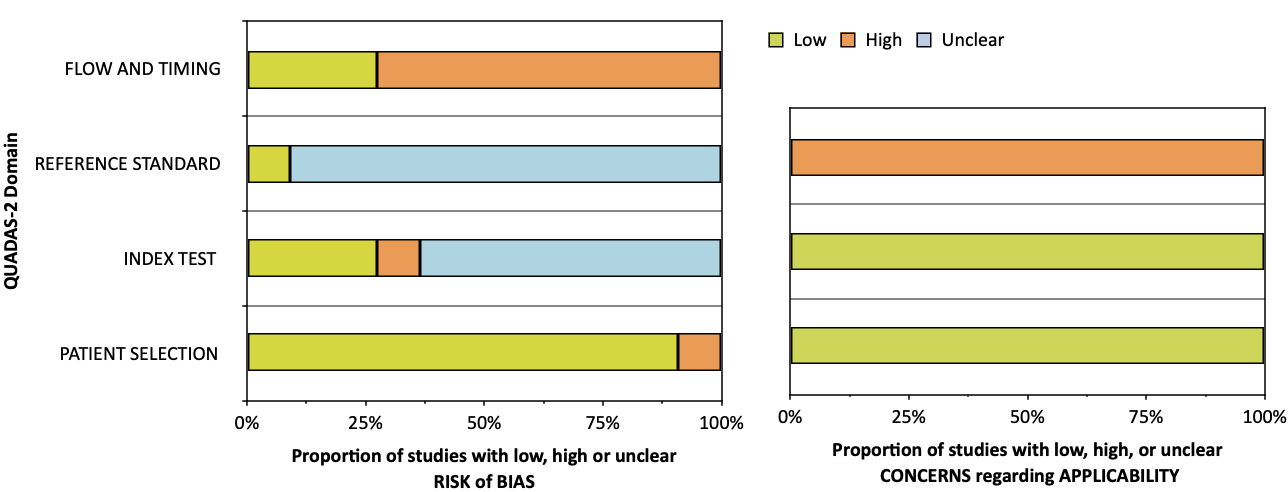


**Supplementary Table 1. Fourfold table values (TP, FP, FN, TN) and corresponding sensitivity and specificity for threshold values of ASL-derived biomarkers in the discrimination between progression and therapy-induced changes.**

| Author and year | ASL Parameter | Threshold | Sensitivity | Specificity | TP | FP | FN | TN |
| --- | --- | --- | --- | --- | --- | --- | --- | --- |
| Liu et al. 2020 | rCBF | UR | 0.75 | 0.929 | 12 | 1 | 4 | 13 |
| Manning et al. 2020 | rCBF | 1.57 | 0.92 | 0.857 | 23 | 1 | 2 | 6 |
|  | CBF | 64.2 ml/100g/min | 1.00 | 0.88 | 25 | 1 | 0 | 6 |
| Ozsunar et al. 2010 | rCBF | 1.3 | 0.92 | 0.50 | 12 | 3 | 1 | 3 |
| Razek et al. 2018 | CBF | 27.8 ml/100g/min | 0.938 | 0.917 | 23 | 1 | 1 | 17 |
| Seeger et al. 2013 | rCBF | 2.18 | 0.539 | 0.846 | 8 | 2 | 6 | 10 |
| Wang et al. 2018 | rCBF | 1.86 | 0.686 | 0.912 | 24 | 3 | 11 | 31 |
|  | CBF | 36.86 ml/100g/min | 0.743 | 0.824 | 26 | 6 | 9 | 28 |
| Xu et al. 2017 | rCBF | 1.11 | 1.00 | 0.75 | 17 | 3 | 0 | 9 |
|  | CBF | 32.325 ml/100g/min | 0.882 | 0.583 | 15 | 5 | 2 | 7 |
| Ye et al. 2015 | rCBF | UR | UR | UR | UR | UR | UR | UR |

UR: unreported

**Supplementary Table 2. Diagnostic accuracy assessment of ASL-derived biomarkers in identifying post-treatment progression in glioma patients.**

| Index | Studies number | Total  *n* | Progression  *n* | Remission  *n* | Sensitivity  [95% CI] | Specificity  [95% CI] |
| --- | --- | --- | --- | --- | --- | --- |
| rCBF | 6 | 204 | 120 | 84 | 0.85  [0.67, 0.94] | 0.83  [0.71, 0.91] |
| rCBF max | 5 | 178 | 106 | 72 | 0.88  [0.71, 0.96] | 0.83  [0.67, 0.92] |
| rCBF mean | 1 | * | * | * | * | * |
| CBF max | 4 | 172 | 101 | 71 | 0.93  [0.73, 0.98] | 0.84  [0.67, 0.93] |

*: impossible pooling due to the limited number of studies. *n*: number of pooled patients; CI: confidence interval.

**Supplementary Table 3. Sensitivity analysis of rCBF studies, showing the effect of removing each individual study on the analysis results.**

| **Removed Record** | **Pooled effect estimate (SMD) [95%CI]** |
| --- | --- |
| Liu et al. 2019 | 1.22 [0.63, 1.81] |
| Manning et al. 2020 | 1.08 [0.76, 1.39] |
| Ozsunar et al. 2010 | 1.36 [0.84,1.89] |
| Seeger et al. 2013 | 1.35 [0.78, 1.91] |
| Wang et al. 2018 | 1.26 [0.61, 1.91] |
| Xu et al. 2017 | 1.32 [0.73, 1.91] |
| Ye et al. 2015 | 1.25 [0.68, 1.82] |
| **SMD of all included studies is 1.25 [0.75, 1.75]** | |

**Supplementary Table 4. Sensitivity analysis of rCBF max studies, showing the effect of removing each individual study on the analysis results.**

| **Removed Record** | **Pooled effect estimate (SMD) [95%CI]** |
| --- | --- |
| Liu et al. 2019 | 1.33 [0.64, 2.03] |
| Manning et al. 2020 | 1.14 [0.80, 1.48] |
| Ozsunar et al. 2010 | 1.50 [0.91,2.09] |
| Wang et al. 2018 | 1.39 [0.61, 2.17] |
| Xu et al. 2017 | 1.45 [0.77, 2.14] |
| Ye et al. 2015 | 1.37 [0.70, 2.03] |
| **SMD of all included studies is 1.35 [0.78, 1.91]** | |

**Supplementary Table 5. Sensitivity analysis of CBF max studies, showing the effect of removing each individual study on the analysis results.**

| **Removed Record** | **Pooled effect estimate (SMD) [95%CI]** |
| --- | --- |
| Razek et al. 2018 | 1.22 [0.78, 1.65] |
| Wang et al. 2018 | 1.71 [0.29, 3.14] |
| Xu et al. 2017 | 1.83 [0.73, 2.94] |
| **SMD of all included studies is 1.56 [0.79, 2.33]** | |
